# Metformin Attenuates Palmitate-Induced Endoplasmic Reticulum Stress, Serine Phosphorylation of IRS-1 and Apoptosis in Rat Insulinoma Cells

**DOI:** 10.1371/journal.pone.0097868

**Published:** 2014-06-04

**Authors:** Laura Simon-Szabó, Márton Kokas, József Mandl, György Kéri, Miklós Csala

**Affiliations:** 1 Department of Medical Chemistry, Molecular Biology and Pathobiochemistry, Semmelweis University, Budapest, Hungary; 2 MTA-SE Pathobiochemistry Research Group, Department of Medical Chemistry, Molecular Biology and Pathobiochemistry, Semmelweis University, Budapest, Hungary; 3 Vichem Chemie Research Ltd., Budapest, Hungary; University of Nebraska Medical Center, United States of America

## Abstract

Lipotoxicity refers to cellular dysfunctions caused by elevated free fatty acid levels playing a central role in the development and progression of obesity related diseases. Saturated fatty acids cause insulin resistance and reduce insulin production in the pancreatic islets, thereby generating a vicious cycle, which potentially culminates in type 2 diabetes. The underlying endoplasmic reticulum (ER) stress response can lead to even β-cell death (lipoapoptosis). Since improvement of β-cell viability is a promising anti-diabetic strategy, the protective effect of metformin, a known insulin sensitizer was studied in rat insulinoma cells. Assessment of palmitate-induced lipoapoptosis by fluorescent microscopy and by detection of caspase-3 showed a significant decrease in metformin treated cells. Attenuation of β-cell lipotoxicity was also revealed by lower induction/activation of various ER stress markers, e.g. phosphorylation of eukaryotic initiation factor 2α (eIF2α), c-Jun N-terminal kinase (JNK), insulin receptor substrate-1 (IRS-1) and induction of CCAAT/enhancer binding protein homologous protein (CHOP). Our results indicate that the β-cell protective activity of metformin in lipotoxicity can be at least partly attributed to suppression of ER stress.

## Introduction

Type 2 diabetes is a global epidemic that has been spread in all countries and threatens a continually growing population. It is a complex metabolic disorder affecting the complete fuel homeostasis including the storage and mobilization of nutrients as well as the control of plasma lipoprotein and sugar levels. Obesity, sedentary lifestyle and unhealthy diet largely increase the risk of the disease. Low metabolic rate and decreased muscle-fat ratio tend to decrease insulin-responsiveness of the target tissues, which is considered as the underlying defect in this type of diabetes [1]. The onset is silent and often remains unrecognized for several years because insulin resistance can be compensated for by enhanced secretion of insulin from the pancreatic β-cells. Reduced metabolic response to insulin results in sustained elevation of blood sugar and free or non-esterified fatty acid (FFA or NEFA) levels due to insufficient utilization of glucose and exaggerated fat mobilization in the adipose tissue, respectively. Glucose and FFA in turn synergistically stimulate insulin secretion [2] and a new steady state can be achieved at higher β-cell activity. Accordingly, the metabolic syndrome and the onset of type 2 diabetes are characterized by simultaneous hyperglycemia and hyperinsulinemia. However, permanently increased concentrations of glucose and/or FFA turned out to be toxic to β-cells, and hence the weaker the tissues respond to insulin the less effectively it is counterbalanced. Aggravation of this derangement results in the exhaustion and death of β-cells, and a substantial shrinkage of the compensatory potential, a key event in the progress of the disease [3].

Viability of β-cells is undoubtedly a major determinant for the development and progress of type 2 diabetes. Contribution of lipotoxicity (i.e. deleterious effects of fatty acids) to β-cell dysfunction and β-cell death has lately come into the focus of interest, and it is now regarded to play a major role in the pathomechanism [4]. Long-chain saturated fatty acids, including palmitate and stearate, induce dominantly apoptotic β-cell death (lipoapoptosis) in culture and isolated islets [5]. Unsaturated fatty acids are usually less toxic or even protective [6]. Although the metabolic background of fatty acid induced damages has not yet been fully elucidated, it became evident that endoplasmic reticulum (ER) stress is a central mediator of lipoapoptosis [7]. The ER functions as a nutrient sensor in the cells, and fuel surplus can induce or facilitate ER stress [8]. Long term exposure to saturated fatty acids was shown to cause ER stress via ER Ca^2+^ depletion [9]. Increased protein load in the ER due to stimulated insulin secretion makes pancreatic β-cells particularly susceptible to this condition.

ER stress triggers the unfolded protein response (UPR), a signaling network of three main branches initiated by three sensors in the ER membrane: inositol-requiring enzyme 1 (IRE1), RNA-dependent protein kinase-like ER kinase (PERK) and activating transcription factor 6 (ATF6) [7]. PERK-dependent phosphorylation of eukaryotic initiation factor, eIF2α decreases the protein load by attenuating general translation. The ATF6-dependent adaptive transcriptional alterations (e.g. induction of ER chaperones) are enhanced by X-box-binding protein 1 (XBP1) transcription factor, which is synthesized upon IRE1-mediated splicing a 26-base fragment from its mRNA. However, the UPR also initiates death signals, which take effect once the stress is prolonged. Induction of CCAAT/enhancer binding protein homologous protein (CHOP) and activation of c-Jun N-terminal kinase (JNK) belong to the major ER-derived pro-apoptotic events. In addition, JNK-dependent serine (307) phosphorylation of insulin receptor substrate-1 (IRS-1) is a key link between ER-stress and insulin resistance. Moreover, insulin resistance within the β-cells is suggested to aggravate the impaired insulin secretion and contribute to cell damage [10].

Prevention or reduction of lipotoxicity induced ER-stress, with special emphasis on JNK activation and serine phosphorylation of IRS-1, in pancreatic β-cells is a promising antidiabetic strategy [11]. Metformin, a widely used insulin sensitizer has been shown to protect HepG2 human hepatoma cell line [12] and human pancreatic islets [13] against lipotoxicity. It has also been reported recently to prevent ER stress induced apoptosis in a mouse β-cell line [14]. The aim of our work was to examine whether attenuation of the ER stress response might play a role in the β-cell protection by metformin in lipotoxicity. Palmitate-induced lipotoxic ER stress and lipoapoptosis were assessed in RINm5F rat insulinoma cells [15]. Our findings revealed a significant reduction in several palmitate-induced UPR events by metformin. Most importantly, the observed decrease in lipoapoptosis can be, at least partly, due to the interference of metformin with lipotoxic JNK activation, IRS-1 serine phosphorylation and CHOP induction.

## Materials and Methods

### Materials Used

Culture medium and supplements were purchased from Life Technologies. Metformin was obtained from Vichem Chemie LTD; palmitate, fatty acid free bovine serum albumin and thapsigargin were purchased from Sigma Aldrich. All other chemicals were of analytical grade.

### Cell Culture Maintenance and Treatment

RINm5F rat insulinoma cells [15] were obtained from ATCC and cultured in complete growth medium: RPMI 1640 medium with 2 mM L-glutamine adjusted to contain 1.5 g/l sodium bicarbonate, 4.5 g/l glucose, 10 mM HEPES and 1 mM sodium pyruvate and supplemented with 10% fetal bovine serum and antibiotics at 37°C in a humidified atmosphere containing 5% CO_2_. Cells were treated with palmitate (500 µM), metformin (10 µM or 100 µM) or thapsigargin (10 µM) for 6 or 8 h starting at 70–80% confluence in 6-well plates (for Western blot and RT-PCR) or in 12-well or 96-well plates (for assessment of cell viability, apoptosis and necrosis). Palmitate was conjugated to fatty acid free bovine serum albumin in 3∶1 molar ratio and incubated at 37°C for an hour prior to addition to the cell culture medium. Untreated control cells received an equal volume of palmitate free vehicle.

### Cell Viability, Apoptosis and Necrosis Detection

Cell viability was assessed by the trypan blue exclusion method [16]. The culture medium was collected and the adherent cells were removed from the surface by trypsine. The trypsinized cells were combined with the supernatant and centrifuged at 200×g for 5 min at room temperature. The cell pellets were re-suspended in fresh medium and 10 µl of cell suspension was mixed with 10 µl 0.4% trypan blue stain. Live and dead (stained) cells were counted using Countess Automated Cell Counter (Invitrogen) according to the manufacturer’s instructions. Cell viability was expressed as the percentage of live cells in the total cell population.

Apoptotic and necrotic cells were detected by using fluorescence microscopy and Annexin-V-FLUOS Staining Kit (Roche) according to the manufacturer’s instructions. Cells with green fluorescence (Annexin V labeling) were considered as apoptotic while those with red or both green and red fluorescence (propidium iodide DNA staining) were considered as necrotic. In each experimental condition, a minimum of 500 cells was counted. The necrosis and apoptosis indexes were calculated as (necrotic or apoptotic cells)/(cells counted)×100. For Western blot or RT-PCR analysis,

### Western Blot Analysis of Cell Lysates

Cells were washed twice with PBS, harvested in 150 µl lysis buffer by scraping and brief vortexing. The lysis buffer contained 0.1% SDS, 5 mM EDTA, 150 mM NaCl, 50 mM Tris, 1% Tween 20, 1 mM Na_3_VO_4_, 1 mM PMSF, 10 mM benzamidine, 20 mM NaF, 1 mM pNPP and protease inhibitor cocktail. The lysates were stored at −20°C until use, and then centrifuged in a benchtop centrifuge (10 min, 10,000×g, 4°C). Protein concentration of the supernatant was measured with Pierce BCA Protein Assay Kit (Thermo Scientific).

Cell lysates (50 µg protein) were electrophoresed on 10% SDS polyacrylamide gels and transferred to PVDF membrane (Millipore). Primary and secondary antibodies were applied overnight at 4°C and for 1 h at room temperature, respectively. Equal protein loading was validated by detection of β-actin as a constitutively expressed reference protein. Horseradish peroxidase (HRP)-conjugated goat polyclonal anti-β-actin (Santa Cruz, sc-1616) antibody was used at 1∶1,000 dilution. Primary antibodies: rabbit anti-P-Ser(307)IRS-1 (A7120) from Assay Biotechnology, rabbit anti-P-Thr(183)/Tyr(185)-JNK (#9251S), rabbit anti-P-eIF2α (#9721L), rabbit anti-eIF2α (#9722S), rabbit anti-P-c-Jun (#9261S), rabbit anti-c-Jun (#9165S), rabbit cleaved caspase-3 (#9661) from Cell Signaling; rabbit anti-CHOP (sc-575), goat anti-GRP78 (sc-1050), rabbit anti-PDI (sc-20132) from Santa Cruz. Secondary antibodies: goat anti-rabbit IgG-HRP (sc-2004), donkey anti-goat IgG-HRP (sc-2020) from Santa Cruz. HRP was detected with chemiluminescence using Western Lightning Plus-ECL (Perkin Elmer).

### Assessment of XBP-1 mRNA Splicing with RT-PCR and Endonuclease Cleavage

Total RNA was purified from the cells by using RNeasy Plus Mini Kit (Quagen) following the manufacturer’s instruction. cDNA was produced by reverse transcription of 0.5–1 µg DNA-free RNA samples using SuperScript III First-Strand Synthesis System for RT-PCR Kit (Invitrogen). Spliced and unspliced XBP-1 sequences (421 or 447 bp, respectively) were amplified by PCR using SY121041268-007 XBP-1 sense (rat) and ST00450236-001 XBP-1 antisense (mouse, rat) primers (Sigma) and iProof High-Fidelity DNA Polymerase Kit (Bio Rad) at thermocycle conditions of 98°C 3 min, 30 cycles of 98°C 10 sec, 57°C 30 sec and 72°C 15 sec and 72°C 10 min final extension. PCR products were purified by PEG precipitation and their concentration was measured with Nanodrop 1000 Spectrophotometer (Thermo Scientific). PstI restriction endonuclease treatment was carried out according to the manufacturer’s instructions. Briefly, 200 ng purified PCR product was digested with FastDigest PstI (Thermo Scientific) for 30 min at 37°C. The amplified sequence of unspliced XBP-1 is cut in two fragments (153 and 294 bp) by PstI while the spliced variant remains uncut [17]. Equal amounts of digested PCR products were resolved by electrophoresis in 2% agarose gel and visualized by EtBR staining.

### Statistics

Western blot results were quantified by densitometry using ImageQuant 5.2 software and are shown as relative band densities normalized to an appropriate reference protein. Data are presented as mean values ± S.D. and were compared using ANOVA with Tukey’s multiple comparison post hoc test. Differences with a P value below 0.05 were considered to be statistically significant.

## Results

### Palmitate-induced Apoptosis in RINm5F Cells

**Figure 1:**
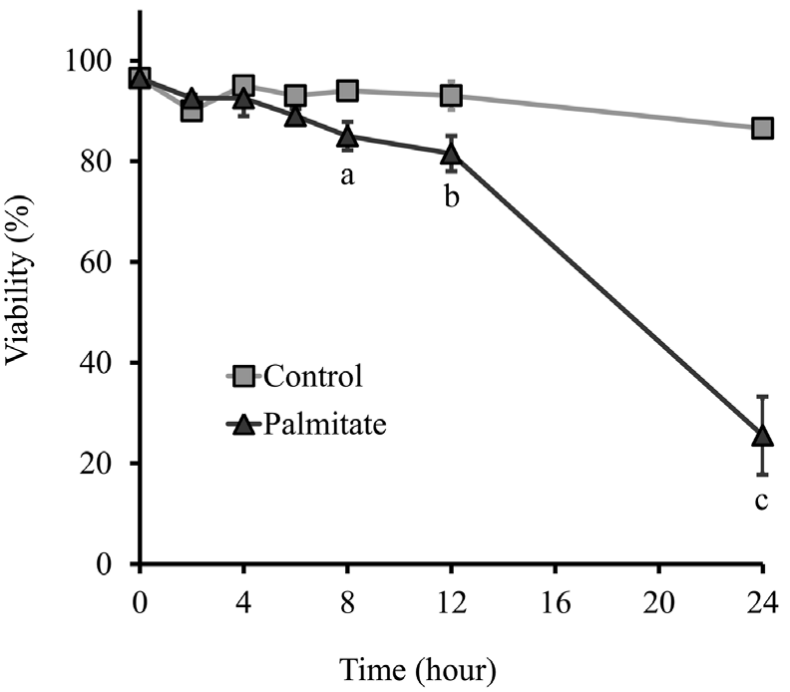
Cell viability. RINm5F rat insulinoma cells were treated with palmitate (500 µM) or vehicle at 70–80% confluence and incubated for various time periods up to 24 h as indicated. Cell viability was assessed by trypan blue exclusion and expressed as the percentage of live cells in the total cell population. Data are presented as mean values ± S.D. of three experiments; ^a^P<0.05, ^b^P<0.01, ^c^P<0.001 v.s. untreated control.

β-Cell protective effect of metformin was tested by assessing lipoapoptosis in RINm5F rat insulinoma cells. Cell death was provoked by the addition of albumin-conjugated palmitate at 500 µM concentration, according to previous studies using the same cell line [15]. Palmitate treatment did not cause a significant change in β-cell viability within the first 6 h; however an accelerating decrease in the number of viable cells was observed in longer incubations and approximately 75% of palmitate-treated cells died within 24 h (Fig. 1). This time course and several previous findings in β-cells [18]–[20] or other cell types [21]–[23] indicated that early lipoapoptosis and the underlying mechanisms could be best investigated at 6–8 h.

**Figure 2:**
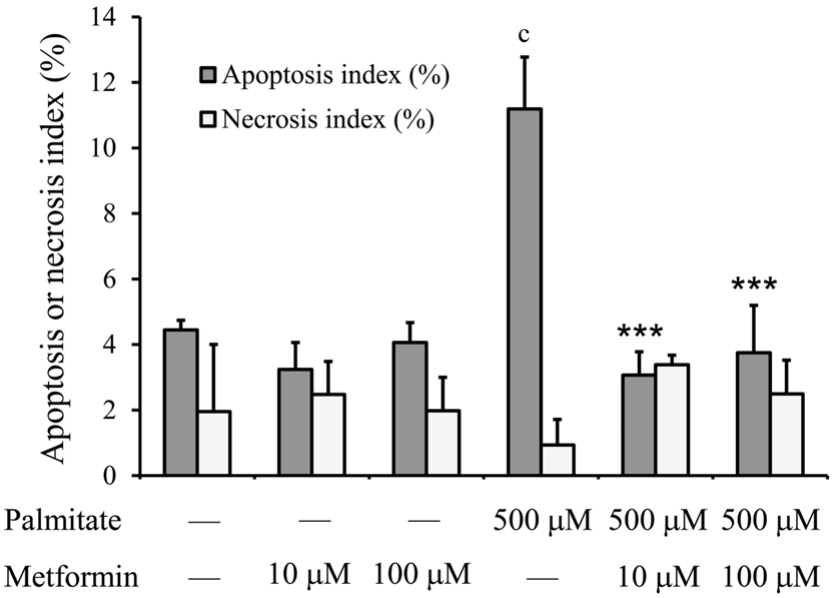
Lipoapoptosis. Insulinoma cells were treated with palmitate (500 µM) and/or metformin (10 µM, 100 µM) at 70–80% confluence. The apoptotic index (number of apoptotic cells/bodies in 100 cells) and necrosis index (necrotic cells in 100 cells) were determined after 6 h by simultaneous Annexin V and propidium iodide (PI) staining and fluorescent microscopy. Annexin V-positive/PI-negative staining was regarded as apoptosis and PI-positive staining as necrosis. Data are presented as mean values ± S.D. of three experiments; ^c^P<0.001 v.s. untreated control; ***P<0.001 v.s. palmitate-treated.

Both apoptosis and necrosis were quantified after Annexin-V staining and fluorescent microscopy. As it is shown in Fig. 2, treatment of RINm5F cells with 500 µM palmitate for 6 h nearly tripled the apoptotic index (11.2%±1.6% vs. 4.4%±0.3%; palmitate treated vs. untreated control; p<0.001; n = 3). Metformin (10 µM or 100 µM) alone did not affect the apoptotic index; nevertheless it completely abolished the pro-apoptotic activity of palmitate when administered simultaneously, i.e. the apoptotic index was reduced to the level of untreated control cells (Fig. 2). Neither metformin nor palmitate had any significant effect on the necrosis index in our experiments (Fig. 2).

### Effect of Metformin on Palmitate-induced ER Stress

**Figure 3:**
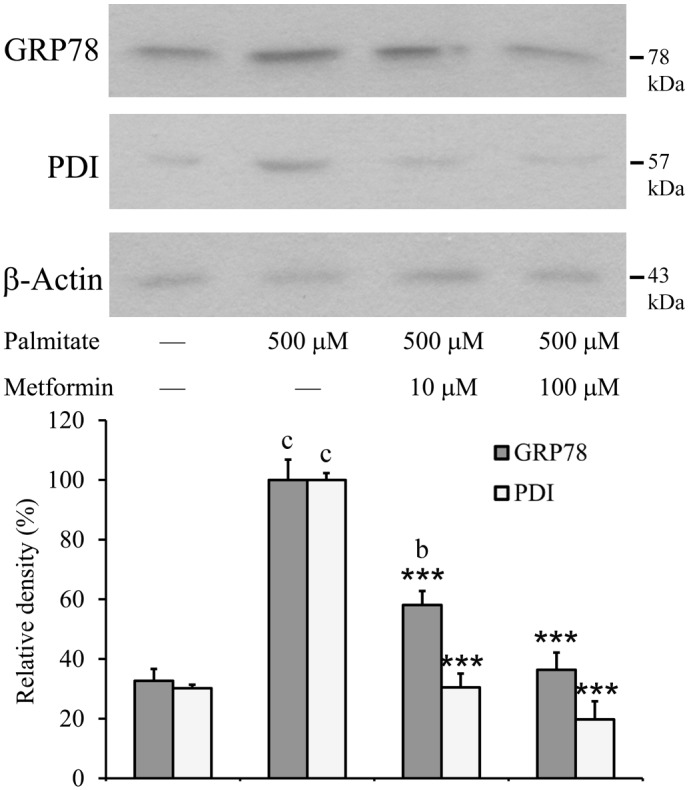
Expression of ER chaperones, GRP78/BiP and PDI. Insulinoma cells were treated with palmitate (500 µM) alone or together with metformin (10 µM, 100 µM) at 70–80% confluence. GRP78 and PDI were detected by Western blot analysis using cell lysates prepared after 8 h. Typical results of three independent experiments are shown. The results were quantified by densitometry and are shown as relative band densities normalized to β-actin as a constitutive reference protein. Data are presented as mean values ± S.D. of three experiments in arbitrary units (palmitate-treated = 100%); ^b^P<0.01,^ c^P<0.001 v.s. untreated control; ***P<0.001 v.s. palmitate-treated.

Induction of ER chaperones is a fundamental element of the UPR and a well-established marker of ER stress. The amount of two major ER chaperones was assessed by Western blot. Both glucose-regulated protein 78 (GRP78) also known as BiP and protein disulfide isomerase (PDI) were largely induced in the palmitate-treated cells compared to controls. This ER chaperone inducing effect of lipotoxicity was markedly counteracted by simultaneous addition of metformin. The expression of BiP and PDI was significantly lower than in the palmitate-treated cells, and 100 µM metformin reduced the amount of both chaperons to the control level (Fig. 3).

### Interference with PERK-initiated Events of the UPR

**Figure 4:**
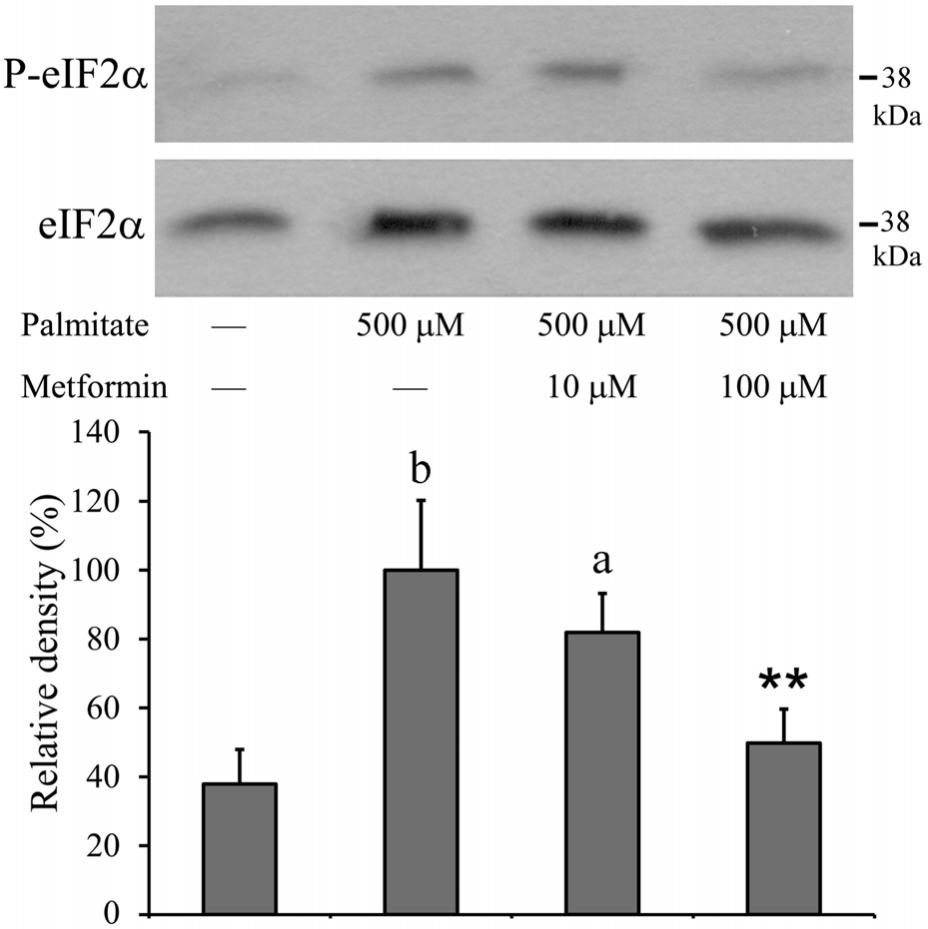
Phosphorylation of eIF2α. Insulinoma cells were treated with palmitate (500 µM) alone or together with metformin (10 µM, 100 µM) at 70–80% confluence. Cell lysates were prepared after 8 h and the phosphorylation and expression level of eIF2α were assessed by Western blot analysis using antibodies specific to phosphorylated (upper panel) and total (lower panel) eIF2α, respectively. Typical results of three independent experiments are shown. The results were quantified by densitometry and are shown as normalized relative band densities. Data are presented as mean values ± S.D. of three experiments in arbitrary units (palmitate-treated = 100%); ^a^P<0.05, ^b^P<0.01 v.s. untreated control; **P<0.01 v.s. palmitate-treated.

PERK is responsible for the attenuation of general translation through phosphorylation of eIF2α. This phenomenon was well detectable in palmitate-treated RINm5F cells by Western blot using a P-eIF2α specific primary antibody (Fig. 4). The amount of phosphorylated eIF2α was approximately 3-times higher in treated v.s. untreated cells, strongly indicating the activation of PERK-initiated events of the UPR. A partial inhibition of eIF2α phosphorylation was observed when palmitate was administered together with metformin. The antidiabetic agent was only effective at higher (100 µM) concentration, and P-eIF2α was still increased to about twice the control level (Fig. 4).

**Figure 5:**
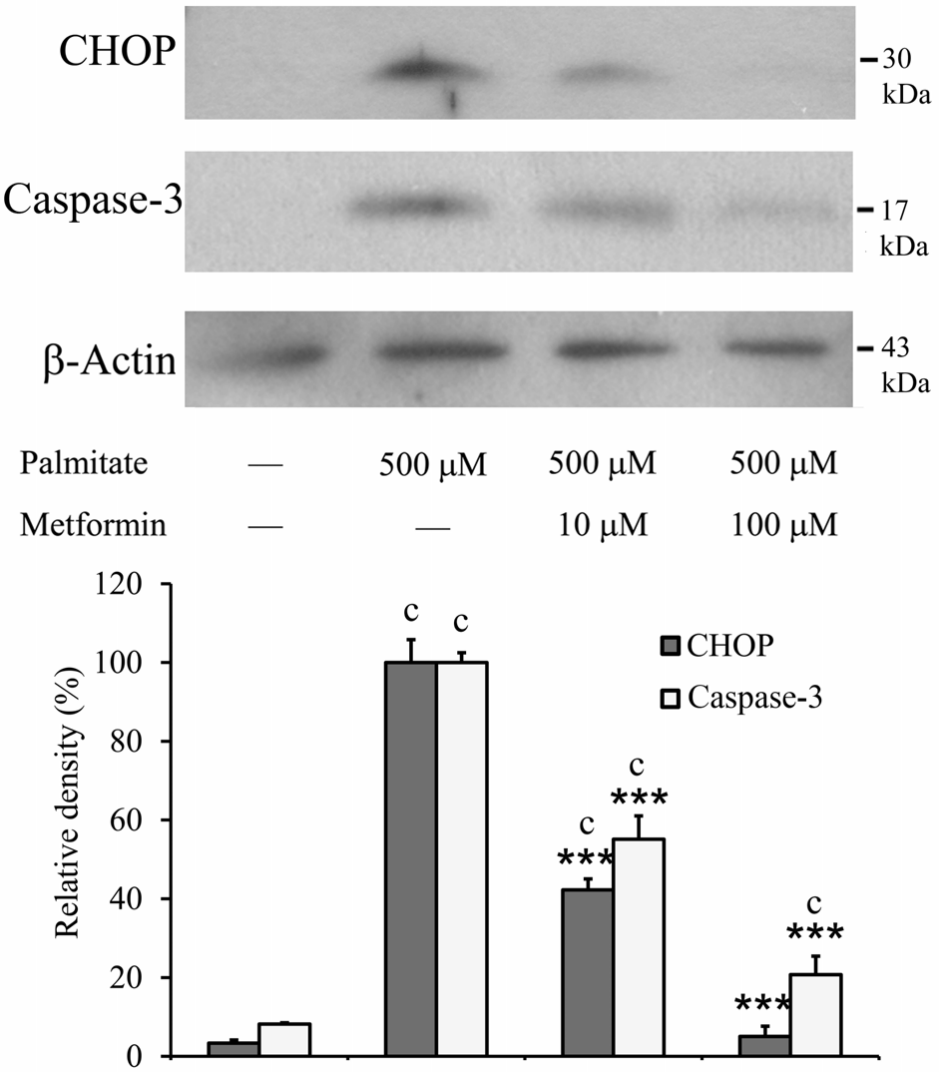
CHOP induction and caspase-3 cleavage. Insulinoma cells were treated with palmitate (500 µM) alone or together with metformin (10 µM, 100 µM) at 70–80% confluence. CHOP and cleaved caspase-3 were detected by Western blot analysis using cell lysates prepared after 8 h. Typical results of three independent experiments are shown. The results were quantified by densitometry and are shown as relative band densities normalized to β-actin as a constitutive reference protein. Data are presented as mean values ± S.D. of three experiments in arbitrary units (palmitate-treated = 100%); ^c^P<0.001 v.s. untreated control; ***P<0.001 v.s. palmitate-treated.

Phosphorylation of eIF2α is known to contribute to the stimulated expression of CHOP, an ER stress specific pro-apoptotic protein. Metformin was found to be effective in moderating the palmitate-dependent CHOP induction. Remarkable (about 30-fold) increase in CHOP expression was only observed when palmitate was added alone. The extent of this induction was approximately halved by 10 µM and essentially abolished by 100 µM metformin as revealed by Western blot analysis (Fig. 5). In connection with this reduction of CHOP expression and in accordance with the observed apoptosis prevention, metformin treatment also effectively counteracted the palmitate-induced activation of caspase-3. Although cleaved caspase-3 was still detectable in metformin-treated cells, its level decreased in parallel with CHOP expression (Fig. 5).

### Modulation of the IRE1 Pathway

**Figure 6:**
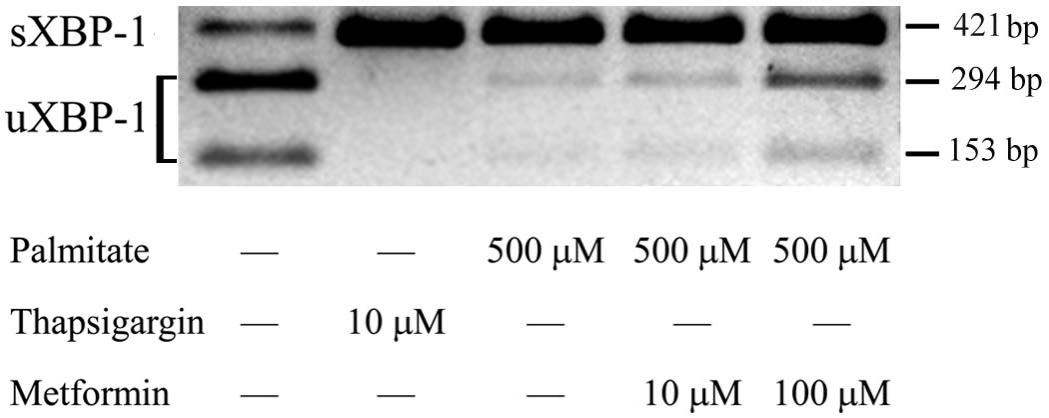
IRE1-dependent splicing of XBP-1 mRNA. Insulinoma cells were treated with thapsigargin (10 µM) as a positive control, palmitate (500 µM) alone or together with metformin (10 µM, 100 µM) at 70–80% confluence. Total RNA was prepared after 8 h and unspliced (uXBP-1) and spliced (sXBP-1) XBP-1 mRNA sequences were amplified by RT-PCR. PstI restriction endonuclease cleavage yields two fragments (153 and 294 bp) from uXBP-1 PCR product while leaves sXBP-1 uncut (421 bp). The products were separated by 2% agarose gel electrophoresis. Typical results of three independent experiments are shown.

IRE1 activation is well represented by the excision of 26 nucleotides from XBP-1 mRNA, which can be visualized by agarose gel electrophoresis after RT-PCR amplification and endonuclease digestion of the affected region. This unconventional splicing was revealed in palmitate-treated as well as in thapsigargin-treated (positive control) RINm5F cells (Fig. 6). A marked increase in the amount of unspliced XBP-1 mRNA demonstrates the concentration dependent antagonistic effect of metformin on palmitate-induced IRE-1 activation. However, large amounts of spliced mRNA can be seen in the palmitate and metformin treated samples, which indicates that this branch of the UPR signaling network is inhibited to a relatively lower extent (Fig. 6).

**Figure 7:**
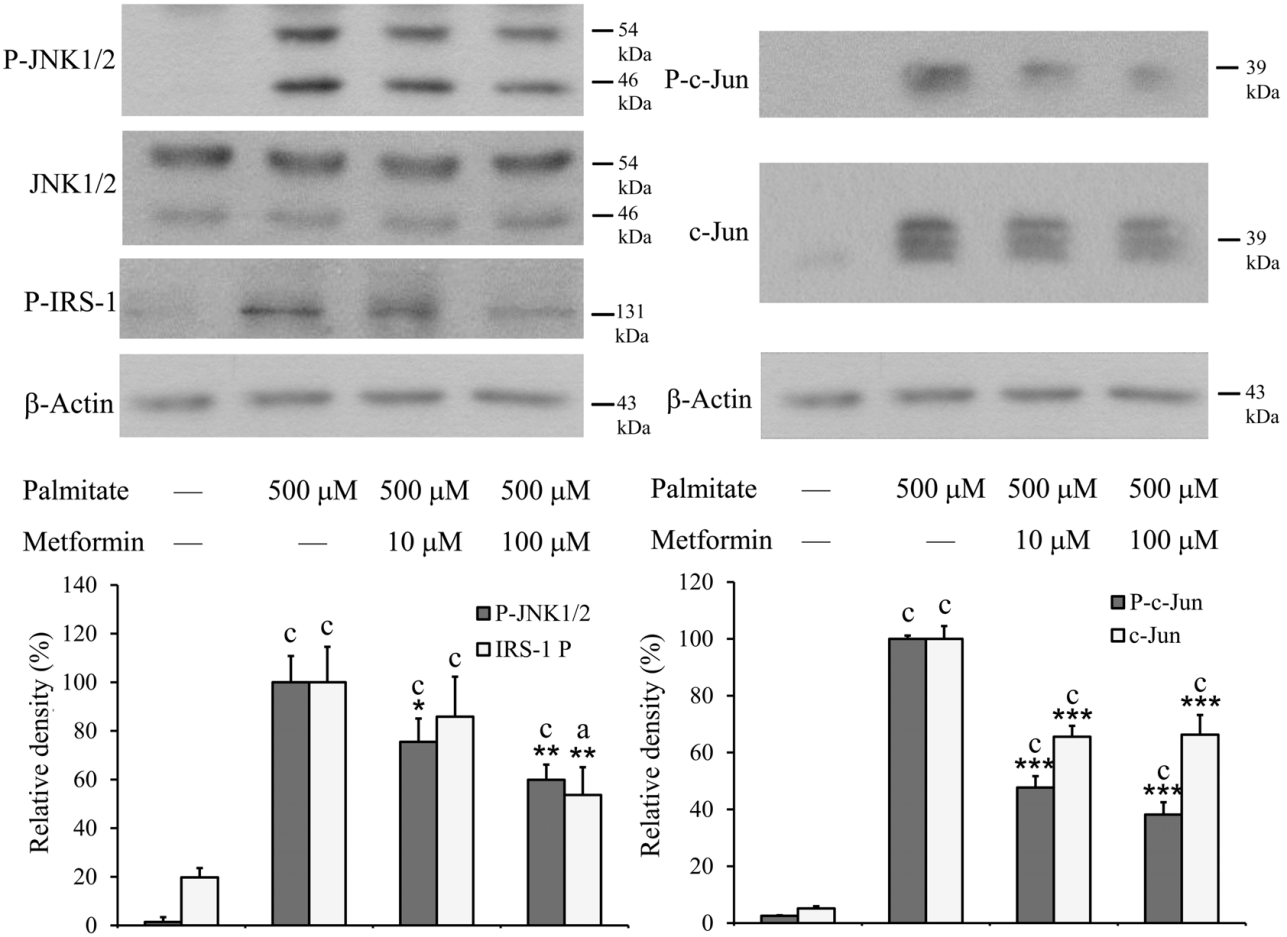
Phosphorylation of JNK, c-Jun and IRS-1. Insulinoma cells were treated with palmitate (500 µM) alone or together with metformin (10 µM, 100 µM) at 70–80% confluence. Total and phosphorylated JNK (two isoforms), phosphorylated (at Ser 307) IRS-1, total and phosphorylated c-Jun were detected by Western blot analysis using cell lysates prepared after 8 h. Typical results of three independent experiments are shown. The results were quantified by densitometry and are shown as relative band densities normalized to β-actin as a constitutive reference protein. Data are presented as mean values ± S.D. of three experiments in arbitrary units (palmitate-treated = 100%); ^a^P<0.05, ^c^P<0.001 v.s. untreated control; *P<0.05, **P<0.01 ***P<0.001 v.s. palmitate-treated.

Phosphorylation of JNK is also mediated by activated IRE-1. The two most important substrates of P-JNK, c-Jun and IRS-1 play important roles in the induction of apoptosis and insulin resistance. Phosphorylation of the two JNK isoforms, JNK1 and 2, c-Jun and IRS-1 (Fig. 7) was detected by immunoblot using the appropriate phosphorylation-specific antibodies. Largely enhanced JNK activation was found in palmitate-treated cells, which was antagonized by metformin in a concentration dependent manner. None of these phosphorylations was completely eliminated but they were all reduced to nearly half of the extent revealed in palmitate-only-treated cells (Fig. 7).

### Exposure of RINm5F to Metformin Only

**Figure 8:**
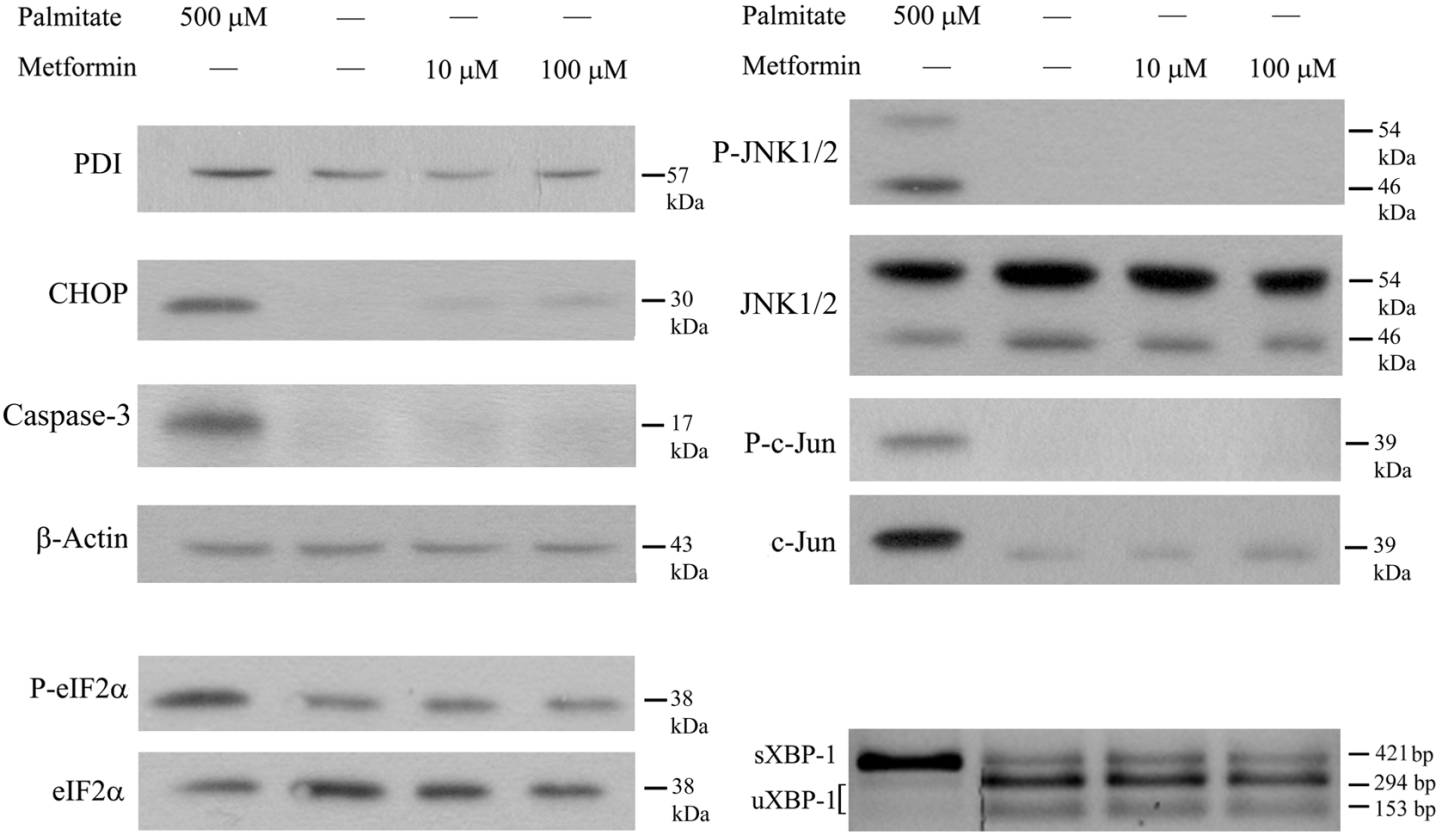
Treatment with metformin only. Insulinoma cells were treated with 500 µM palmitate (positive control) or metformin (10 µM, 100 µM) at 70–80% confluence. PDI, CHOP, cleaved caspase-3, Total and phosphorylated eIF2α, total and phosphorylated JNK (two isoforms), total and phosphorylated c-Jun and β-actin as a constitutive reference protein were detected by Western blot analysis using cell lysates prepared after 8 h. Total RNA was also prepared after 8 h and unspliced (uXBP-1) and spliced (sXBP-1) XBP-1 mRNA sequences were amplified by RT-PCR. PstI restriction endonuclease cleavage yields two fragments (153 and 294 bp) from uXBP-1 while leaves sXBP-1 uncut (421 bp). The products were separated by 2% agarose gel electrophoresis. Typical results of three independent experiments are shown.

As it was shown in Fig. 2, metformin treatment in the absence of palmitate did not affect the intensity of apoptosis or necrosis in RINm5F cells. The possible effect of metformin on the investigated parameters of the UPR including caspase-3 activation and c-Jun phosphorylation was also tested in our experimental conditions. Palmitate treatment was applied as a positive control. Metformin (10 or 100 µM) did not cause any statistically significant change in the expression level of PDI, CHOP, eIF2α, c-Jun or JNK; in the phosphorylation of the latter three proteins or in the activation of caspase-3 (Fig. 8).

## Discussion

Insulin secretion in the pancreatic β-cells is stimulated in response to nutrient abundance during the fed state. The primary regulator is plasma glucose but its stimulatory effect is also enhanced by FFAs and amino acids [3]. Increased insulin level normally achieves the acceleration of glucose consumption in various tissues (liver, skeletal muscle and adipose tissue). It also shifts both protein and triglyceride turnovers toward the synthesis, and thereby favours the utilization of plasma amino acids and FFAs, too [24].

Overfeeding increases the challenge to β-cells, which need to synthesize and secrete more insulin. The balance can be maintained as long as the main metabolic tissues (liver, skeletal muscle and adipose tissue) obey and increase their contribution. However, insulin-responsiveness and the fuel utilizing capacity of the body largely depend on genetic predisposition and environmental factors. The lack of physical activity and the related obesity are considered as important causes of insulin resistance, the primary disorder in type 2 diabetes [1]. It leads to elevation of plasma FFA level, which further aggravates insulin resistance [25]. The vicious cycle may culminate in β-cell death and decreased insulin producing capacity. Prevention of type 2 diabetes or hindrance of its progression by a variety of lifestyle changes and drugs is likely dependent on β-cell protection. Moreover, it became evident that counteraction of β-cell failure is a promising therapeutic strategy. It is therefore, important to investigate the current anti-diabetic agents from this aspect [11].

Deleterious effects of elevated FFA levels on β-cells and the role of lipotoxicity in diabetes were discovered long ago [26]. Further investigation of the phenomenon revealed the involvement of lipotoxic ER stress [7]. One of the primary adaptive mechanisms of ER stress is the attenuation of general translation through phosphorylation of eIF2α, which can decrease the insulin secreting capacity of β-cells. In addition, prolonged and severe ER stress induces apoptosis, and thereby contributes to the reduction of β-cell mass. ER stress dependent activation of JNK is one of the main pro-apoptotic events, which also favors insulin resistance by means of Ser-phosphorylation of IRS-1 [27]. Although this latter mechanism was primarily studied in the main metabolic tissues (liver, skeletal muscle etc.), it turned out to be important in the derangement of the control of insulin secretion in the β-cells [10].

Metformin is one of the leading anti-diabetic drugs. Its most appreciated effect is the improvement of insulin responsiveness; however, its direct β-cell protective effect was also demonstrated long ago [13]. Although metformin has been shown to increase AMP-activated protein kinase activity, its molecular target has not been unequivocally elucidated [28]. Our results show that metformin significantly reduces lipotoxicity in a β-cell line. Palmitate-induced apoptosis and some major events of the underlying ER stress response (i.e. PDI and Grp78 induction and eIF2α phosphorylation) were practically abolished by metformin in a concentration-dependent manner. Interestingly, the IRE1 pathway of the UPR (i.e. unconventional splicing of XBP-1 mRNA and JNK, c-Jun and IRS-1phosphorylation) showed a markedly lower extent of inhibition. Most importantly, however, induction of the pro-apoptotic transcription factor CHOP and generation of the cleaved effector caspase-3 were also largely repressed by metformin, which can underlie the observed decrease in palmitate-induced apoptosis. The apparent discrepancy between the completely abrogated apoptosis and the less pronounced JNK, c-Jun and IRS-1 phosphorylations can be explained by the convergence of the UPR pathways. In contrast to the phosphorylation of JNK, c-Jun and IRS-1, which are clearly associated to the IRE1 pathway, CHOP induction is due to a coordinated action of all the three branches of the UPR. The expression of CHOP is controlled simultaneously by three major ER-stress-related transcription factors (ATF6, the PERK-dependent ATF4 and the IRE1-dependent XBP-1) [29]. Therefore, the remaining activity of only one signaling pathway might be unable to maintain elevated CHOP levels and stimulated apoptosis.

Similar effects of metformin, i.e. cell protection and prevention of lipotoxic ER stress have been observed also in HepG2 human hepatoma cell line [12]. In line with our findings, the phenomenon was accompanied by a reduced Ser-phosphorylation of IRS-1, which might contribute to insulin-sensitizing in hepatocytes. Our findings demonstrating these effects of metformin in a rat insulinoma cell line have a great importance since β-cell protection and maintenance of insulin sensitivity in the β-cells are of particular significance in the prevention and treatment of diabetes.

Preventive effect of metformin on ER stress-induced apoptosis in NIT-1 cells (a mouse pancreatic beta cell line) has been recently reported [14]. ER stress was provoked by the SERCA inhibitor thapsigargin and, unlike palmitate-induced ER stress in our study, it was not found to be counteracted. Nevertheless, the consequent apoptosis as well as JNK activation and IRE-1 phosphorylation were efficiently reduced by metformin. These effects were attributed to AMP-activated protein kinase and phosphatidylinositol-3 kinase activation. These data suggest that, besides the evident amelioration of ER stress, additional mechanisms might contribute to the abrogation of lipoapoptosis and the massive suppression of JNK activation in our experiments.

In summary, our findings further support the β-cell protective potential of metformin. Attenuation of lipoapoptosis in RINm5F rat insulinoma cell line can be attributed to modulation of palmitate-induced ER stress response in general. Decreased activation of JNK is of special importance because of its role in both the induction of apoptosis and the development of insulin resistance. Besides the partly restored insulin sensitivity, an enhanced durability of β-cells might underlie the improved prognosis of metformin treated diabetic patients.

## References

[1] Ye J (2013) Mechanisms of insulin resistance in obesity. Front Med 7: 14–24. 23471659 10.1007/s11684-013-0262-6 PMC3936017

[2] Itoh Y, Kawamata Y, Harada M, Kobayashi M, Fujii R, et al (2003) Free fatty acids regulate insulin secretion from pancreatic beta cells through GPR40. Nature 422: 173–176. 12629551 10.1038/nature01478

[3] Fu Z, Gilbert ER, Liu D (2013) Regulation of insulin synthesis and secretion and pancreatic Beta-cell dysfunction in diabetes. Curr Diabetes Rev 9: 25–53. 22974359 PMC3934755

[4] Marchetti P, Bugliani M, Boggi U, Masini M, Marselli L (2012) The pancreatic beta cells in human type 2 diabetes. Adv Exp Med Biol 771: 288–309. 23393686 10.1007/978-1-4614-5441-0_22

[5] Shimabukuro M, Zhou YT, Levi M, Unger RH (1998) Fatty acid-induced beta cell apoptosis: a link between obesity and diabetes. Proc Natl Acad Sci U S A 95: 2498–2502. 9482914 10.1073/pnas.95.5.2498 PMC19389

[6] Welters HJ, Tadayyon M, Scarpello JH, Smith SA, Morgan NG (2004) Mono-unsaturated fatty acids protect against beta-cell apoptosis induced by saturated fatty acids, serum withdrawal or cytokine exposure. FEBS Lett 560: 103–108. 14988006 10.1016/S0014-5793(04)00079-1

[7] Cnop M, Igoillo-Esteve M, Cunha DA, Ladriere L, Eizirik DL (2008) An update on lipotoxic endoplasmic reticulum stress in pancreatic beta-cells. Biochem Soc Trans 36: 909–915. 18793160 10.1042/BST0360909

[8] Mandl J, Meszaros T, Banhegyi G, Hunyady L, Csala M (2009) Endoplasmic reticulum: nutrient sensor in physiology and pathology. Trends Endocrinol Metab 20: 194–201. 19349192 10.1016/j.tem.2009.01.003

[9] Cunha DA, Hekerman P, Ladriere L, Bazarra-Castro A, Ortis F, et al (2008) Initiation and execution of lipotoxic ER stress in pancreatic beta-cells. J Cell Sci 121: 2308–2318. 18559892 10.1242/jcs.026062 PMC3675788

[10] Accili D (2004) Lilly lecture 2003: the struggle for mastery in insulin action: from triumvirate to republic. Diabetes 53: 1633–1642. 15220184 10.2337/diabetes.53.7.1633

[11] Bonora E (2008) Protection of pancreatic beta-cells: is it feasible? Nutr Metab Cardiovasc Dis 18: 74–83. 18096375 10.1016/j.numecd.2007.05.004

[12] Kim DS, Jeong SK, Kim HR, Kim DS, Chae SW, et al (2010) Metformin regulates palmitate-induced apoptosis and ER stress response in HepG2 liver cells. Immunopharmacol Immunotoxicol 32: 251–257. 20038265 10.3109/08923970903252220

[13] Lupi R, Del Guerra S, Fierabracci V, Marselli L, Novelli M, et al (2002) Lipotoxicity in human pancreatic islets and the protective effect of metformin. Diabetes 51 Suppl 1S134–137. 11815472 10.2337/diabetes.51.2007.s134

[14] Jung TW, Lee MW, Lee YJ, Kim SM (2012) Metformin prevents endoplasmic reticulum stress-induced apoptosis through AMPK-PI3K-c-Jun NH2 pathway. Biochem Biophys Res Commun 417: 147–152. 22138650 10.1016/j.bbrc.2011.11.073

[15] Beeharry N, Chambers JA, Green IC (2004) Fatty acid protection from palmitic acid-induced apoptosis is lost following PI3-kinase inhibition. Apoptosis 9: 599–607. 15314288 10.1023/B:APPT.0000038039.82506.0c

[16] O’Brien R, Gottlieb-Rosenkrantz P (1970) An automatic method for viability assay of cultured cells. J Histochem Cytochem 18: 581–589. 5449983 10.1177/18.8.581

[17] Williams BL, Lipkin WI (2006) Endoplasmic reticulum stress and neurodegeneration in rats neonatally infected with borna disease virus. J Virol 80: 8613–8626. 16912310 10.1128/JVI.00836-06 PMC1563873

[18] Baldwin AC, Green CD, Olson LK, Moxley MA, Corbett JA (2012) A role for aberrant protein palmitoylation in FFA-induced ER stress and beta-cell death. Am J Physiol Endocrinol Metab 302: E1390–1398. 22436701 10.1152/ajpendo.00519.2011 PMC3378068

[19] Oh YS, Lee YJ, Kang Y, Han J, Lim OK, et al (2013) Exendin-4 inhibits glucolipotoxic ER stress in pancreatic beta cells via regulation of SREBP1c and C/EBPbeta transcription factors. J Endocrinol 216: 343–352. 23257266 10.1530/JOE-12-0311

[20] Sommerweiss D, Gorski T, Richter S, Garten A, Kiess W (2013) Oleate rescues INS-1E beta-cells from palmitate-induced apoptosis by preventing activation of the unfolded protein response. Biochem Biophys Res Commun 441: 770–776. 24189472 10.1016/j.bbrc.2013.10.130

[21] Cho H, Wu M, Zhang L, Thompson R, Nath A, et al (2013) Signaling dynamics of palmitate-induced ER stress responses mediated by ATF4 in HepG2 cells. BMC Syst Biol 7: 9. 23339444 10.1186/1752-0509-7-9 PMC3557202

[22] Olsen GS, Hansen BF (2002) AMP kinase activation ameliorates insulin resistance induced by free fatty acids in rat skeletal muscle. Am J Physiol Endocrinol Metab 283: E965–970. 12376323 10.1152/ajpendo.00118.2002

[23] Tao JL, Wen YB, Shi BY, Zhang H, Ruan XZ, et al (2012) Endoplasmic reticulum stress is involved in podocyte apoptosis induced by saturated fatty acid palmitate. Chin Med J (Engl) 125: 3137–3142. 22932195

[24] Combettes-Souverain M, Issad T (1998) Molecular basis of insulin action. Diabetes Metab 24: 477–489. 9932214

[25] Frohnert BI, Jacobs DR, Jr., Steinberger J, Moran A, Steffen LM, et al.. (2013) Relation between Serum Free Fatty Acids and Adiposity, Insulin Resistance and Cardiovascular Risk Factors from Adolescence to Adulthood. Diabetes. 10.2337/db12-1122 PMC3749355 23670973

[26] Unger RH (1995) Lipotoxicity in the pathogenesis of obesity-dependent NIDDM. Genetic and clinical implications. Diabetes 44: 863–870. 7621989 10.2337/diab.44.8.863

[27] Aguirre V, Uchida T, Yenush L, Davis R, White MF (2000) The c-Jun NH(2)-terminal kinase promotes insulin resistance during association with insulin receptor substrate-1 and phosphorylation of Ser(307). J Biol Chem 275: 9047–9054. 10722755 10.1074/jbc.275.12.9047

[28] Musi N, Hirshman MF, Nygren J, Svanfeldt M, Bavenholm P, et al (2002) Metformin increases AMP-activated protein kinase activity in skeletal muscle of subjects with type 2 diabetes. Diabetes 51: 2074–2081. 12086935 10.2337/diabetes.51.7.2074

[29] Kim I, Xu W, Reed JC (2008) Cell death and endoplasmic reticulum stress: disease relevance and therapeutic opportunities. Nat Rev Drug Discov 7: 1013–1030. 19043451 10.1038/nrd2755

